# Impact of the COVID-19 pandemic on reports of violence: a temporal analysis, Minas Gerais state, 2014-2022

**DOI:** 10.1590/S2237-96222026v35e20250720.en

**Published:** 2026-07-20

**Authors:** Dalmo Abrantes Figueiredo, Karen Duarte Moreira, Larissa Rocha Manhães Alves, Paloma Dornas de Castro, Waneska Alexandra Alves, Eulilian Dias de Freitas

**Affiliations:** 1 Universidade Federal de Juiz de Fora Departamento de Medicina Governador Valadares MG Brazil Universidade Federal de Juiz de Fora, Departamento de Medicina, Governador Valadares, MG, Brazil

**Keywords:** Violence, Life Cycle, COVID-19, Notifiable Health Conditions Information System, Pandemics, Violencia, Ciclo de Vida, COVID-19, Sistema de Información de Enfermedades de Declaración Obligatoria, Pandemias

## Abstract

**Objectives:**

To characterize reports of interpersonal violence and their temporal trend, and to analyze the impact of the COVID-19 pandemic on reports of this type of violence in Minas Gerais state, from 2014 to 2022.

**Methods:**

Time series study using Notifiable Health Conditions Information System data. Reports of interpersonal violence in Minas Gerais, 2014-2022, were analyzed using absolute and relative frequency, mean and standard deviation, and incidence of violence by sex and stage of life. The Prais-Winsten generalized linear regression model was used to estimate the impact of COVID-19 on reporting, taking a 5% significance level.

**Results:**

268,377 reports of violence were recorded during the period. The predominant subgroups were women (70.7%) and mixed-race individuals (45.5%). Physical violence was the most frequently reported (55.5% in women, 67.6% in men). Sexual violence against children stood out (37.8% in girls, 19.6% in boys), as did neglect/abandonment among elderly people (13.9% in women, 12.0% in men). The pandemic impacted the incidence of violence in all subgroups, even those with a rising trend in the pre-pandemic period. During the pandemic, incidence rates remained below previous levels, represented by negative and statistically significant β2 coefficients.

**Conclusion:**

Women and mixed-race individuals were the most affected by interpersonal violence in Minas Gerais. Temporal trends in violence reporting were impacted during the pandemic compared to the pre-pandemic period in the stages of life studied. The importance of strategies for maintaining surveillance of health problems during times of health crisis was demonstrated.

## Resumen

**Objetivos:**

Caracterizar los informes de violencia interpersonal y su tendencia temporal, y analizar el impacto de la pandemia de COVID-19 en los informes de este tipo de violencia en el estado de Minas Gerais, de 2014 a 2022.

**Métodos:**

Estudio de series temporales con datos del Sistema de Información de Enfermedades de Declaración Obligatoria. Se analizaron los informes de violencia interpersonal en Minas Gerais, 2014-2022, utilizando frecuencia absoluta y relativa, media y desviación estándar, e incidencia de violencia por sexo y ciclo de vida. Se empleó el modelo de regresión lineal generalizada de Prais-Winsten para estimar el impacto de la COVID-19 en los informes, con un nivel de significancia del 5%.

**Resultados:**

Se registraron 268.377 informes de violencia durante el período. Los subgrupos predominantes fueron mujeres (70,7%) e individuos mestizos (45,5%). La violencia física fue la más frecuentemente reportada (55,5% en mujeres, 67,6% en hombres). La violencia sexual contra los niños fue un fenómeno destacado (37,8% en niñas, 19,6% en niños), al igual que el abandono o la negligencia entre las personas mayores (13,9% en mujeres, 12,0% en hombres). La pandemia impactó la incidencia de violencia en todos los subgrupos, incluso en aquellos con una tendencia creciente en el periodo prepandémico. Durante la pandemia, las incidencias se mantuvieron por debajo de los niveles previos, representadas por coeficientes β2 negativos y estadísticamente significativos.

**Conclusión:**

Las mujeres y las personas de raza mixta fueron las más afectadas por la violencia interpersonal en Minas Gerais. Las tendencias temporales en la notificación de violencia se vieron afectadas durante la pandemia en comparación con el periodo prepandémico en los ciclos de vida estudiados. Se demostró la importancia de las estrategias para mantener la vigilancia de los problemas de salud durante las crisis sanitarias.

**Ethical aspects**

This research used public domain anonymized databases.

## Introduction

In 1996, the 49th World Health Assembly declared that violence is a global public health problem. It is capable of generating short- and long-term repercussions for victims, affecting people of all age groups [1,2]. The term violence refers to the intentional use of force or power, whether threatened or actual, “against oneself, against another person, or against a group or community”, with the potential to “result in injury, death, psychological harm, maldevelopment or deprivation” [2]. Interpersonal violence is defined as the intentional use of force or power against an individual or group of individuals, including physical, sexual, psychological violence and neglect [3].

In Brazil, violence in general is the sixth leading cause of hospitalizations, and mortality due to this condition has been increasing in recent years, especially among younger people. Furthermore, both unintentional injuries and those related to violence account for 10% of years lived with disability [1]. In 2023, in Brazil, the homicide mortality rate (the ultimate form of interpersonal violence) was 21.2 per 100,000 inhabitants. In Minas Gerais state, in the same year, there were 12.9 deaths per 100,000 inhabitants. The highest rate was found in Amapá state (57.4 per 100,000 inhabitants) and the lowest in São Paulo state (6.4 per 100,000 inhabitants) [4].

The consequences of interpersonal violence vary according to the type of violence suffered and the victim’s stage of life [5]. Children and adolescents are primarily victims of sexual, physical and emotional violence. Global prevalence of interpersonal violence for this population, between 2010 and 2022, was estimated at 17%. It is mainly perpetrated by people close to the victims [6]. Among females, there is a predominance of physical, sexual and psychological violence, mainly committed by intimate partners [7,8]. At the extremes of age, neglect by caregivers becomes a notable form of violence. Among males, across all stages of life, there is higher incidence of physical violence, especially in public places [8].

The effects of interpersonal violence, regardless of stage of life, are vast and lasting, in addition to increasing the risk of infectious diseases, reproductive health problems, mental disorders and chronic non-communicable diseases [3]. At the community level, the consequences include loss of workforce, overburdened health services and increased demands on the judicial system [9].

In 2020, COVID-19, caused by the SARS-CoV-2 virus, quickly became an international public health emergency, generating a global crisis. The pandemic resulted in the fragmentation of social capital, family structures and livelihoods around the world [10]. Social distancing and quarantine measures, adopted to contain the spread of the virus, resulted in school closures [11], home confinement, economic stress and changes in family routines [9]. This scenario altered the dynamics of coexistence of people or groups vulnerable to interpersonal violence with their aggressors, potentially reflecting in its reporting [12].

In Brazil, there was a change in the temporal trend of reports of sexual violence against children and adolescents after the start of the pandemic, with a 3% increase (p-value 0.001) between 2017 and 2021 [13]. Specifically in Paraná state, regarding the profile of sexual violence against adult women during the intrapandemic period, 70% of cases occurred at home; in 46% of cases the perpetrator was known to the victim [14].

Regarding violence against the elderly during the pandemic in the state of São Paulo, those aged 75 or older were most frequently victims of neglect or abandonment, which accounted for 56% of reports [15]. Although there are specific studies, analyses of the potential for change in reporting trends of this problem, by stage of life, comparing the period before and during the pandemic, are incipient.

It is relevant to understand the indirect effects of public health emergencies, such as the COVID-19 pandemic, on health outcomes. The objectives of this study were to characterize reports of interpersonal violence and their temporal trend, and to analyze the impact of the COVID-19 pandemic on reports of this type of violence in Minas Gerais state, from 2014 to 2022.

## Methods

### Study design

This is an epidemiological, analytical, time-series study of reported cases of interpersonal violence, according to stage of life, that occurred in Minas Gerais, between 2014 and 2022.

### Setting

This study used non-nominal secondary data extracted from the Brazilian Unified Health System Notifiable Health Conditions Information System and provided by the Minas Gerais State Health Department on August 22, 2022.

### Participants

The study population consisted of individuals who were victims of interpersonal violence in Minas Gerais, regardless of sex, age, race/skin color or education level, during the study period.

All records of reported interpersonal violence in Minas Gerais, between January 1, 2014, and August 12, 2022, were included, provided their International Classification of Diseases, 10th revision code fell between X85 and Y099. Records of self-inflicted injuries and cases that did not occur within the state were excluded.

### Study variables

The variables used in the study for descriptive analyses were: sex (male, female), race/skin color (White, Black, Asian, mixed-race, Indigenous), type of violence suffered (physical, psychological, sexual, neglect/abandonment, torture, other [financial, legal, child labor, human trafficking]) and stage of life, stratified into children (0-9 years), adolescents (10-19 years), adults (20-59 years) and elderly (60 years or more).

When building the model, average incidence of interpersonal violence in two periods (pre-pandemic and pandemic) was taken as the dependent variable. The independent variables were time (discrete, in months since the beginning of the observations), pandemic (dichotomous, taking a value of 0 in the pre-pandemic period and 1 in the pandemic period) and COVID-19 (discrete, in months in the pandemic period and assigning 0 to the pre-pandemic period).

The two time segments were comprised as follows:

1) pre-pandemic period, which included reports of cases that occurred from July 1, 2014 to March 31, 2020; and
2) pandemic period, which included reports of interpersonal violence dated from April 1, 2020 to May 31, 2022.

The period used to begin the time series was defined based on Ministry of Health Ordinance No. 1,271, dated June 6, 2014. This ordinance included domestic, sexual, and/or other forms of violence on the mandatory reporting list. It determined immediate reporting of cases of sexual violence and attempted suicide at the municipal level. Therefore, July 2014, that is, the month following the promulgation of the Ordinance, was taken as the first month of the model.

The data for June, July and August 2022 were considerably lower than in previous months. Therefore, in order to maintain a conservative approach, the end of the series was set for May 31, 2022, the most complete update available at the time the manuscript was written.

### Statistical methods

Initially, a descriptive analysis was conducted, using measures of absolute frequency, proportions and measures of central tendency. Student’s t-test was used to compare means. Monthly incidence rates of interpersonal violence were calculated for each stage of life and by sex by dividing the number of reports for each subgroup in each month by the total number of residents exposed that year, for each stage of life and sex, multiplied by 100,000. We used the Brazilian Institute of Geography and Statistics 2018 annual population projection for Minas Gerais. In order to calculate average incidence in the pre-pandemic and pandemic periods, the monthly incidence rates for each period were added together and divided by the total number of months in each period.

In order to ascertain the impacts of the COVID-19 pandemic on reporting of interpersonal violence according to stage of life, we used a segmented regression model for interrupted time series, as proposed by Wagner et al. [16]. The Prais-Winsten generalized linear regression method was chosen to estimate the model in order to circumvent serial autocorrelation of the residuals [17,18]. This resulted in:


$$Y_{i}=\beta_{0}+\beta_{1}\text{Time}+\beta_{2}\text{Pandemic}+\beta_{3}\text{COVID19}$$

where:

Y^i^ is the incidence of interpersonal violence; 

Time, is the count variable that indicates the time in months since the start of the observations; 

Pandemic, is the dichotomous variable that assigns a value of 1 during the COVID-19 pandemic and 0 before it (impact); 

COVID19, is the discrete variable that counts time in months during the pandemic, assigning a value of 0 to the remaining months (trend);

​​β​ _0_​​​, is the intercept before the pandemic;

​​β​ _1_​​​, is the change in ​​Y​ i​​​ over time;

​​β​ _2_​​​, is the impact on the incidence of interpersonal violence with effect from the onset of the pandemic; and

​​β​ _3_​​​, the change in ​​Y​ i​​​ added to ​​β​ 1 ​​​over time during the pandemic.

A 5% significance level was established for all statistical tests. The data were analyzed using Stata 12 software.

## Results

During the nine years studied, 268,337 reports of interpersonal violence were recorded. In the analysis, 70.7% of victims were female, a result that was repeated for all stages of life. The 9.2% incompleteness rate for the race/skin color field stood out. Among the records that did contain this information, 45.6% of victims of interpersonal violence were of mixed-race (Table 1).

**Table 1:** Absolute (n) and relative (%) frequencies of reports of interpersonal violence in each stage of life, by sex, race/skin color and type of violence. Minas Gerais, 2014-2022 (n=268,337)

	Children	Adolescents	Adults	Elderly	Total
	n (%)	n (%)	n (%)	n (%)	n (%)
**Sex **					
Male	9,444 (39.7)	18,617 (33.9)	41,098 (24.3)	9,398 (45.3)	78,557 (29.3)
Female	14,365 (60.3)	36,292 (66.1)	127,669 (75.7)	11,416 (54.9)	189,742 (70.7)
**Race/skin color**					
White	7,140 (30.0)	16,202 (29.5)	54,931 (32.5)	8,517 (40.9)	86,790 (32.3)
Black	2,296 (9.6)	6,343 (11.6)	20,462 (12.1)	2,390 (11.5)	31,491 (11.7)
Mixed-race	11,117 (46.7)	25,994 (47.3)	76,930 (45.6)	8,175 (39.3)	122,216 (45.6)
Other	325 (1.4)	587 (1.1)	2,027 (1.2)	213 (1.0)	3,152 (1.2)
No information	2,937 (12.3)	5,791 (10.5)	14,438 (8.6)	1,522 (7.3)	24,688 (9.2)
Total	23,815 (100.0)	54,917 (100.0)	168,788 (100.0)	20,817 (100.0)	268,337 (100.0)
**Type of violence **					
**Physical violence **					
Female	5,696 (27.9)	25,388 (48.6)	112,631 (61.7)	7,428 (43.2)	151,143 (55.5)
Male	5,243 (39.9)	15,922 (68.5)	38,136 (77.0)	7,318 (58.1)	66,619 (67.6)
**Psychological violence **					
Female	3,982 (19.5)	12,179 (23.3)	50,922 (27.9)	5,138 (29.9)	72,221 (26.5)
Male	2,518 (19.1)	3,844 (16.5)	7,682 (15.5)	2,483 (19.7)	16,527 (16.8)
**Sexual violence **					
Female	7,739 (37.9)	10,923 (20.9)	7,930 (4.4)	470 (2.7)	27,062 (9.9)
Male	2,587 (19.7)	1,232 (5.3)	437 (0.9)	43 (0.3)	4,299 (4.4)
**Violence through neglect/abandonment **					
Female	2,178 (10.7)	1,154 (2.2)	842 (0.5)	2,406 (14.0)	6,580 (2.4)
Male	2,166 (16.5)	853 (3.7)	521 (1.1)	1,521 (12.1)	5,061 (5.1)
**Other types of vi^o^lencea **					
Female	835 (4.1)	2,583 (5.0)	10,112 (5.5)	1,752 (10.2)	15,282 (5.6)
Male	643 (4.9)	1,384 (6.0)	2,749 (5.6)	1,236 (9.8)	6,012 (6.1)
**Total listed types of vi^o^lenceb **					
Female	20,430 (100.0)	52,227 (100.0)	182,437 (100.0)	17,194 (100.0)	272,288 (100.0)
Male	13,157 (100.0)	23,235 (100.0)	49,525 (100.0)	12,601 (100.0)	98,518 (100.0)

^a^Sum of the following types of violence: other, torture, financial, legal, child labor, human trafficking; ^b^A single report can contain more than one type of violence.

Physical violence was the most frequent type among females (55.5%), followed by psychological violence (26.5%). This situation was repeated for all stages of life, except for female children, for whom sexual violence was the most frequent (37.9%). Physical violence was also the most reported type (67.7%) among males and followed the same pattern at all stages of life. Higher frequencies of neglect/abandonment were observed in the extreme stages of life, i.e. among children and the elderly (Table 1).

Across all stages of life, there was a noticeable shift in trends in interpersonal violence following the onset of the COVID-19 pandemic (Figure 1). Comparison of the means and standard deviations of incidence rates in the pre-pandemic and pandemic periods confirmed the falling trend across all stages of life, for both males and females (p-value>0.050 in all categories) (Table 2).

**Table 2:** Mean and standard deviation (SD) of the incidence of reported interpersonal violence in the pre-pandemic and pandemic periods (per 100,000 individuals), in each life cycle, by sex. Minas Gerais, 2014-2022 (n=268,337)

	Pre-pandemic period	Pandemic period	
	Mean (SD)	Mean (SD)	Difference (%)^a^
**Children **			
Female	10.98±1.59	9.76±1.58	11.1
Male	7.24±1.09	5.10±0.86	29.6
Total	9.07±1.19	7.38±1.11	18.6
Adolescents			
Female	25.15±2.67	17.54±2.18	30.3
Male	12.87±1.74	6.87±1.09	35.6
Total	18.88±1.95	12.08±1.33	36.0
Adults			
Female	22.45±1.88	15.86±1.70	29.4
Male	7.04±0.72	5.49±0.94	22.0
Total	14.74±1.21	10.66±1.27	27.7
**Elderly **			
Female	7.80±1.12	4.66±0.54	40.3
Male	7.68±0.99	4.57±0.52	40.5
Total	7.75±0.91	4.62±0.42	40.4

^a^T-test performed to compare means, p-value>0.050 in all categories.

**Figure 1:**
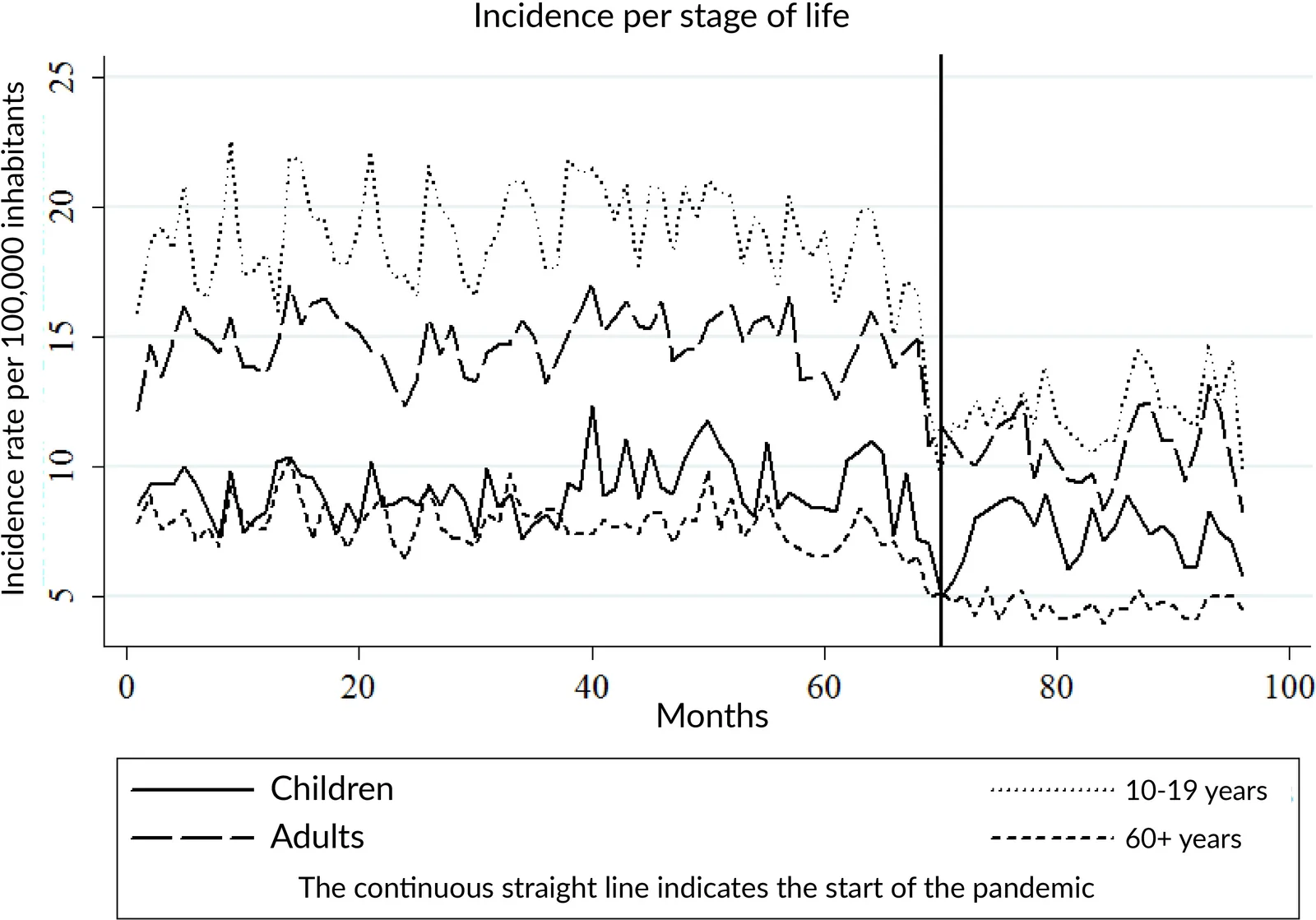
Temporal trend of monthly incidence of interpersonal violence (per 100,000 inhabitants), by stage of life. Minas Gerais, 2014-2022 (n=268,337)

The adjusted linear Prais-Winsten model allowed us to verify two dimensions of average incidence of interpersonal violence: level and trend. The COVID-19 pandemic acted as a temporal segmentation of the incidence of interpersonal violence, allowing a comparison between the pre-pandemic and pandemic periods in the aforementioned dimensions. In the pre-COVID-19 pandemic period, a rising trend was observed in the incidence of interpersonal violence against female children (0.03 reported cases per 100,000 inhabitants; p-value<0.050), while the trend was downward for male adolescents (-0.03 cases per 100,000 inhabitants; p-value<0.050) and the elderly (-0.02 cases per 100,000 inhabitants; p-value<0.050). Incidence of interpersonal violence showed a stationary trend for the other stages of life and by sex (Table 3).

**Table 3:** Interpersonal violence incidence rate slope coefficients and 95% confidence intervals (95%CI) in the pre-COVID-19 pandemic and COVID-19 pandemic periods in each stage of life, by sex. Minas Gerais, 2014-2022 (n=268,337)

Stage of life	Pre-pandemic period		Pandemic period	
	​​𝜷​ 1​​​^a^ (95%CI)	Trend	​​𝜷​ 2​​​^b^ (95%CI)	​​𝜷​ 3​​​^c^ (95%CI)
Children				
Female	0.03 (0.01; 0.05)^d^	Rising	-2.10 (-3.78; -0.43)^d^	-0.03 (-0.12; 0.06)
Male	-0.014 (-0.03; 0.01)	Stationary	-1.60 (-2.85; -0.36)^d^	-0.01 (-0.08; 0.06)
Total	0.0085 (-0.01; 0.03)	Stationary	-1.85 (-3.26; -0.44)^d^	-0.02 (-0.10; 0.06)
Adolescents				
Female	0.02 (-0.02; 0.05)	Stationary	-8.55 (-11.30; -5.81)^d^	0.02 (-0.13; 0.17)
Male	-0.03 (-0.06; -0.01)^d^	Falling	-5.21 (-6.88; -3.55)^d^	0.06 (-0.03; 0.15)
Total	-0.01 (-0.04; 0.02)	Stationary	-6.72 (-8.78; -4.67)^d^	0.03 (-0.08; 0.15)
Adults				
Female	-0.00 (-0.04; 0.03)	Stationary	-5.02 (-7.41; -2.63)^d^	-0.09 (-0.22; 0.05)
Male	-0.01 (-0.02; 0.00)	Stationary	-1.02 (-1.94; -0.10)^d^	-0.01 (-0.06; 0.05)
Total	-0.01 (-0.03; 0.02)	Stationary	-2.95 (-4.54; -1.36)^d^	-0.05 (-0.14; 0.04)
**Elderly **				
Female	-0.02 (-0.03; -0.00)^d^	Falling	-2.44 (-3.52; -1.36)^d^	0.01 (-0.05; 0.07)
Male	-0.02 (-0.03; -0.01)^d^	Falling	-2.26 (-3.08; -1.44)^d^	-0.01 (-0.05; 0.04)
Total	-0.02 (-0.03; -0.01)^d^	Falling	-2.35 (-3.18; -1.53)^d^	0.00 (-0.04; 0.05)

^a^Series trend in the pre-COVID-19 pandemic period; ^b^Measure of the immediate impact of the COVID-19 pandemic, how much mean incidence of interpersonal violence changed (- decrease; + increase); ^c^Measure of the change in the trend during the pandemic. The trend during the pandemic should be interpreted as the sum of β_1_+β_3_; ^d^p-value<0.050.

During the COVID-19 pandemic period, a decrease in reported cases of interpersonal violence was observed across all stages of life, representing a shift in the incidence of interpersonal violence, shown in the table by the negative ​β​_2_ coefficient (shift in ​β​_0_; p-value<0.050). The most significant decreases were in the adolescent category (-8.55 cases per 100,000 inhabitants; p-value<0.050) and the adult category (-5.02 cases per 100,000 inhabitants; p-value<0.050), both occurring in females. The lowest decrease occurred in the adult male subgroup (-1.02 cases per 100,000 inhabitants).

The drop in reported cases continued throughout the COVID-19 pandemic, as the trends did not undergo statistically significant changes in any of the stages of life (​​​β​_3_ not significant), regardless of the pre-pandemic trend (Table 3).

## Discussion

The objective of this study was to characterize reports of interpersonal violence and analyze their temporal trend between 2014 and 2022, as well as the impact of the COVID-19 pandemic on this issue in Minas Gerais. The findings indicated that the largest proportion of reports were of violence against females and mixed-race individuals. The most reported type of violence was physical violence, for both males and females, across all stages of life. An exception was the prevalence of sexual violence against female children, more so than any other type. The onset of the COVID-19 pandemic marked a change in the temporal trend of monthly interpersonal violence incidents, which reduced the reporting of this issue to a greater or lesser degree across all stages of life.

Violence against women is more pronounced in patriarchal societies, which restrict women in different areas of life, hindering their empowerment and autonomy. This situation perpetuates and naturalizes the view of women as inferior and more fragile beings in relation to men, making them more vulnerable to episodes of violence [19]. The higher proportion of victims of mixed race demonstrates the intersectional nature of violence, in which gender, race/skin color, socioeconomic status and other vulnerabilities interact, strengthening the cycle of violence [20]. In Brazil, in 2021, people of Black and mixed-race/skin color, analyzed together, presented risk of violent death two to three times greater than those of White race/skin/color [4,21].

The type of violence to which individuals are at risk varies according to sex and stage of life. Physical violence crosses stages of life differently. While women are mainly victims of domestic violence, notably from intimate partners, men are exposed to violence in external environments, often related to risky behaviors such as alcohol and drug use, and pursuit of status and power [1]. Sexual violence is of a marked gender character, whereby women are the main victims. Objectification of women, coupled with the sense of belonging and dominance, characteristics of a patriarchal society like that found in Brazil, makes them vulnerable right from childhood [19].

In this study, the highest frequencies of neglect/abandonment were observed in the extreme stages of life, which corroborates the hypothesis that power imbalance in relationships is among the causes of interpersonal violence [22]. Children and the elderly are, for the most part, dependent on third-party care for daily activities, in addition to the financial dependence that may coexist [22,23]. Added to this is the fact that seeking medical assistance and reporting aggression among this subgroup is limited. Furthermore, violent episodes may be denied by the victims themselves, for fear of the intra-family and social repercussions they may cause [19]. This emphasizes the need for public policies aimed at preventing violence, with lasting strategies that seek, beyond punishment, to educate for a culture of peace and a society with equal rights.

Several studies on violence, encompassing the period of the COVID-19 pandemic, have shown that this circumstance changed the trend of cases of violence. The results vary according to the methodology [24], the study population [22,25] and the data source [26]. In this study, it was possible to observe that, in all stages of life, both in males and females, average incidence of interpersonal violence before the COVID-19 pandemic was higher than the average during the pandemic (Table 2). At least two factors can explain these results. On the one hand, there may have been a real decrease in cases of violence, although in Brazil violence against women increased from 9.8 to 19.2 per 100,000 women from 2020 to 2023 [20]. On the other hand, there may have been changes in the reporting profile of cases during the pandemic.

Throughout the critical period of the pandemic, the guidance was that health services should only be accessed in urgent cases. Health centers, especially primary care centers, were restructured to meet the demands of the new disease [27]. In the case of violence that occurred at home, social distancing measures facilitated the aggressor’s control over their victims, which made it difficult for them to access their support network and seek help [22]. Regarding children and adolescents, the closure of educational institutions, such as schools and daycare centers, may also have hindered detection of victims of child violence by education professionals, since these workers play an important role in identifying suspected cases [22,27].

As for violence that occurs outside the home, which mainly affects young and adult men, social distancing imposed during the COVID-19 pandemic may have contributed to reducing physical violence [24]. Interaction between these factors, coupled with the fact that there have been no changes in victim protection policies, or significant cultural changes, highlights the possibility that case underreporting was a determining factor in the drop in interpersonal violence incidence rates.

This study demonstrated unchanged trends in the incidence of interpersonal violence during the pandemic period. That is, even after the easing of social distancing, with the adaptation of health services and educational institutions to meet new demands, the reopening of commerce, etc., the trends remained unchanged for subgroups that had previously shown a rising or falling incidence trend, only at lower levels. This result demonstrates that the impacts can be long-lasting and that the pandemic may be yet another agent that makes violence invisible.

This study used secondary data obtained primarily from health services. Therefore, it was not possible to guarantee that data collection was standardized, nor that it represented all cases of interpersonal violence. It is acknowledged that duplications may have occurred. Additionally, the race/skin color variable showed 7% to 12% incompleteness, depending on the stage of life; therefore, data related to it should be interpreted with caution. Despite the limitations inherent to the data source, the Notifiable Health Conditions Information System has been consistently used in epidemiological studies and has demonstrated good validity [26,28,29].

The study design did not enable causality to be inferred, but it did elucidate associations that support the formulation of causal hypotheses to be confirmed in subsequent studies. Generalization of the results should be done with caution, since only reported cases occurring in Minas Gerais were included, which may not be representative of the Brazilian population as a whole. The inclusion of control variables (existence of public policies, local differences in access to healthcare, and regional inequalities) could refine the model and is therefore a possibility for future studies. Furthermore, studies that qualitatively address how the pandemic impacted family and community dynamics can contribute to understanding the ways in which violence persists throughout life, as well as inform discussions on public policies to address this problem.

The conclusion is reached that the COVID-19 pandemic impacted violence reporting heterogeneously across different stages of life, revealing divergent patterns according to gender and age group. This demonstrated that interpersonal violence crosses different stages of life in diverse ways, making certain population subgroups more vulnerable to specific types of violence.

The COVID-19 pandemic was capable of altering the patterns of interpersonal violence incidence, as calculated from interpersonal violence reports in this study. These findings corroborated the importance of health services being prepared for public health emergencies. Furthermore, they highlighted the need for public policies that strengthen reporting channels and victim protection mechanisms, considering the specificities of each stage of life.

## Data Availability

The database and analysis codes used in this research are available at: https://doi.org/10.48331/SCIELODATA.UMERVN [30].
