# Supplementary material for: Impact of the COVID-19 pandemic on reports of violence: a temporal analysis, Minas Gerais state, 2014-2022
Source: Epidemiol Serv Saude. 2026 Jul 20;35:e20250720. doi: 10.1590/S2237-96222026v35e20250720.en (PMC13686299; doi:10.1590/S2237-96222026v35e20250720.en)
Supplement: Translation

# Translation: Impacto da pandemia de covid-19 nas notificações de violência: análise temporal, Minas Gerais, 2014-2022

**DOI:** 10.1590/S2237-96222026v35e20250720.pt

**Authors:** Dalmo Abrantes Figueiredo, Karen Duarte Moreira, Larissa Rocha Manhães Alves, Paloma Dornas de Castro, Waneska Alexandra Alves, Eulilian Dias de Freitas

**Affiliations:** 1 Universidade Federal de Juiz de Fora Departamento de Medicina Governador Valadares MG Brasil Universidade Federal de Juiz de Fora, Departamento de Medicina, Governador Valadares, MG, Brasil

**Keywords:** Violência, Ciclo de Vida, COVID-19, Sistema de Informação de Agravo de Notificação, Pandemias

## Abstract

**Objetivos:**

Caracterizar as notificações de violência interpessoal e sua tendência temporal e analisar o impacto da pandemia de covid-19 nas notificações por esse agravo em Minas Gerais, de 2014 a 2022.

**Métodos:**

Estudo de série temporal, que utilizou dados do Sistema de Informação de Agravos de Notificação. Analisaram-se notificações de violência interpessoal em Minas Gerais, 2014-2022, por meio de frequência absoluta e relativa, média e desvio-padrão e incidência de violência, por sexo e ciclo de vida. Utilizou-se o modelo de regressão linear generalizada de Prais-Winsten para estimar o impacto da covid-19 nas notificações, com significância de 5%.

**Resultados:**

Foram registradas 268.377 notificações de violência no período. Os subgrupos predominantes foram mulheres (70,7%) e pessoas pardas (45,5%). Violência física foi a mais notificada (55,5% em mulheres, 67,6% em homens). Destacaram-se violência sexual em crianças (37,8% em meninas, 19,6% em meninos) e negligência/abandono entre pessoas idosas (13,9% em mulheres, 12,0% em homens). A pandemia impactou a incidência de violência em todos os subgrupos, mesmo naqueles com tendência de crescimento no período pré-pandêmico. Durante o período pandêmico, as incidências se mantiveram aquém dos níveis anteriores, representadas pelo coeficiente de β2 negativos e estatisticamente significativos.

**Conclusão:**

Mulheres e pessoas pardas foram as mais atingidas pela violência interpessoal em Minas Gerais. As tendências temporais de notificação da violência foram impactadas no período pandêmico em relação ao pré-pandêmico nos ciclos de vida estudados. Demonstrou-se a importância de estratégias de manutenção da vigilância de agravos em tempos de crise sanitária.

**Aspectos éticos**

Esta pesquisa utilizou bancos de dados de domínio público e anonimizados.

## Introdução

Em 1996, a 49^a^ Assembleia Mundial de Saúde declarou que a violência constitui um problema de saúde pública global. Ela é capaz de gerar repercussões em curto e longo prazos para as vítimas, afetando pessoas de todas as faixas etárias [1,2]. O termo violência diz respeito à intencionalidade do uso da força ou poder, seja por ato cometido, seja por ameaça “contra si próprio, contra outra pessoa, ou contra um grupo ou uma comunidade”, com potencial de “resultar em lesão, morte, dano psicológico, deficiência de desenvolvimento ou privação” [2]. Define-se violência interpessoal pelo uso intencional de força ou de poder contra um indivíduo ou grupo de indivíduos, incluindo violência física, sexual, psicológica e negligência [3]. 

No Brasil, a violência em geral é a sexta maior causa de hospitalizações, e a mortalidade pelo agravo tem crescido nos últimos anos, principalmente entre os mais jovens. Além disso, tanto lesões não intencionais quanto as relacionadas à violência são responsáveis por 10% dos anos vividos com deficiência [1]. Em 2023, no Brasil, a taxa de mortalidade por homicídio (último grau da violência interpessoal) foi 21,2 por 100 mil habitantes. Já em Minas Gerais, no mesmo ano, foram 12,9 mortes por 100 mil habitantes. A maior taxa foi encontrada no Amapá (57,4 por 100 mil habitantes) e a menor em São Paulo (6,4 por 100 mil habitantes) [4]. 

As consequências da violência interpessoal variam de acordo com o tipo de violência sofrida e do ciclo de vida da vítima [5]. Crianças e adolescentes são vítimas principalmente de violência sexual, física e emocional. A prevalência global de violência interpessoal para essa população no mundo, entre 2010 e 2022, foi estimada em 17%. Ela é perpetrada principalmente por pessoas próximas às vítimas [6]. Entre o sexo feminino, há a predominância de violência física, sexual e psicológica, cometida principalmente por parceiro íntimo [7,8]. Nos extremos de idade, ganha notoriedade a negligência por parte de quem cuida como forma de violência. Entre pessoas do sexo masculino, em todos os ciclos de vida, há a incidência maior de violência física, principalmente em via pública [8].

Os efeitos da violência interpessoal, independentemente do ciclo de vida, são vastos e perduráveis, além de aumentarem o risco de doenças infecciosas, problemas de saúde reprodutiva, transtornos mentais e doenças crônicas não transmissíveis [3]. No nível comunitário, as consequências incluem perda de força de trabalho, sobrecarga dos serviços de saúde e aumento das demandas do sistema judiciário [9].

Em 2020, a covid-19, causada pelo vírus Sars-CoV-2, rapidamente se tornou emergência de saúde pública internacional, o que gerou uma crise global. A pandemia resultou na fragmentação do capital social, das estruturas familiares e dos meios de subsistência ao redor do mundo [10]. Medidas de distanciamento social e quarentena, que foram adotadas para conter a propagação do vírus, resultaram em fechamento de escolas [11], confinamento domiciliar, estresse econômico e mudança na rotina familiar [9]. Esse cenário alterou a dinâmica de convivência de pessoas ou grupos vulneráveis à violência interpessoal com seus agressores, potencialmente refletindo nas notificações desse agravo [12].

No Brasil, houve mudança na tendência temporal das notificações de violência sexual contra crianças e adolescentes após o início da pandemia, com aumento de 3% (p-valor 0,001) entre 2017 e 2021 [13]. Especificamente no Paraná, em se tratando do perfil de violência sexual contra mulheres adultas no período intrapandemia, 70% dos casos ocorreram no domicílio; em 46% dos casos, o agressor era conhecido da vítima [14]. 

Sobre a violência contra pessoas idosas durante a pandemia, no estado de São Paulo, aquelas com 75 anos ou mais foram vítimas mais frequentemente de negligência ou abandono, que chegou a 56% dos relatos [15]. Embora existam estudos pontuais, análises sobre o potencial de mudança nas tendências de notificação desse agravo, por ciclo de vida, comparando o período antes e durante a pandemia, são incipientes.

É relevante entender os efeitos indiretos das emergências de saúde pública, por exemplo, a pandemia de covid-19, nos desfechos em saúde. Os objetivos deste estudo foram caracterizar as notificações de violência interpessoal e sua tendência temporal e analisar o impacto da pandemia de covid-19 nas notificações por esse agravo em Minas Gerais, de 2014 a 2022.

## Métodos

### Desenho do estudo

Trata-se de estudo epidemiológico, analítico, de série temporal das notificações de violência interpessoal, por ciclos de vida, ocorridas em Minas Gerais, entre 2014 e 2022.

### Contexto

Este estudo utilizou dados secundários não nominais extraídos do Sistema de Informação de Agravos de Notificação do Sistema Único de Saúde e fornecidos pela Secretaria Estadual de Saúde de Minas Gerais em 22 de agosto de 2022.

### Participantes

Foram consideradas como população de estudo pessoas vítimas de violência interpessoal ocorrida em Minas Gerais, independentemente de sexo, idade, raça/cor da pele e escolaridade, durante o período pesquisado.

Foram incluídos todos os registros de notificações de violência interpessoal, por local de ocorrência, em Minas Gerais, entre 1º de janeiro 2014 e 12 de agosto de 2022, cujo código da Classificação Internacional de Doenças, 10ª revisão, estivesse contido entre X85 e Y099. Excluíram-se os registros de lesão autoprovocada e os casos não ocorridos no estado.

### Variáveis do estudo

As variáveis utilizadas no estudo para as análises descritivas foram: sexo (masculino, feminino), raça/cor da pele (branca, preta, amarela, parda, indígena), tipo de violência sofrida (física, psicológica/moral, sexual, negligência/abandono, tortura, outra [financeira, legal, trabalho infantil, tráfico humano]) e ciclos de vida, estratificados em crianças (0-9 anos), adolescentes (10-19 anos), adultos (20-59 anos) e pessoas idosas (60 anos ou mais).

Para a construção do modelo, considerou-se variável dependente a incidência média de violência interpessoal em dois períodos (pré-pandêmico e pandêmico). As variáveis independentes foram tempo (discreta, em meses desde o início das observações), pandemia (dicotômica, assumindo valor 0 no período pré-pandêmico e 1 no período pandêmico) e covid-19 (discreta, em meses no período pandêmico e atribuindo-se 0 ao pré-pandêmico).

Os dois segmentos de tempo foram constituídos da seguinte forma:

1) período pré-pandêmico, que compreendeu as notificações ocorridas de 1º julho de 2014 a 31 de março de 2020; e
2) período pandêmico, que incluiu as notificações de violência interpessoal datadas de 1º de abril de 2020 a 31 de maio de 2022.

O período utilizado para o início da série temporal foi definido com base na Portaria nº 1.271, de 6 de junho de 2014, do Ministério da Saúde. Nela, foram incluídas as violências doméstica, sexual e/ou outras na lista de notificação compulsória. O documento estabeleceu a notificação imediata para os casos de violência sexual e tentativa de suicídio em âmbito municipal. Por isso, julho de 2014, isso é, o mês subsequente à promulgação da Portaria, foi considerado o primeiro do modelo.

Os dados referentes a junho, julho e agosto de 2022 apresentavam-se expressivamente inferiores aos meses anteriores. Dessa forma, para manter uma postura conservadora, o fim da série foi definido em 31 de maio de 2022, atualização mais completa à época da elaboração do manuscrito.

### Métodos estatísticos

Inicialmente, conduziu-se a análise descritiva, com medidas de frequência absoluta, proporções e medidas de tendência central. O teste t de Student foi utilizado para comparar as médias. Foram calculadas as taxas de incidência de violência interpessoal mensal, para cada ciclo de vida e sexo, dividindo-se o número de notificações de cada subgrupo, em cada mês, pelo total de residentes expostos naquele ano, de cada ciclo de vida e sexo, multiplicado por 100 mil. Foi utilizada a projeção populacional anual, edição de 2018, do Instituto Brasileiro de Geografia e Estatística para Minas Gerais. Para o cálculo da incidência média nos períodos pré-pandêmico e pandêmico, somaram-se as incidências mensais de cada período, dividindo-as pelo total de meses que compõem cada período.

Para verificar os impactos da pandemia de covid-19 sobre as notificações de violência interpessoal por ciclo de vida, foi utilizado um modelo de regressão segmentada para séries temporais interrompidas conforme proposto por Wagner e colaboradores [16]. Para estimá-lo, optou-se pelo método de regressão linear generalizada de Prais-Winsten, a fim de contornar a autocorrelação serial dos resíduos [17,18]. Tem-se portanto:


$$Y_{i}=\beta_{0}+\beta_{1}\text{Tempo}+\beta_{2}\text{Pandemia}+\beta_{3}\text{COVID19}$$

em que:

Y^i^ é a incidência de violência interpessoal; 

Tempo, a variável de contagem que indica o tempo em meses desde o início das observações; 

Pandemia, a variável dicotômica que assume valor 1 durante a pandemia de covid-19 e 0 antes dela (impacto); 

COVID19, a variável discreta que conta o tempo em meses durante a pandemia, atribuindo valor 0 para os demais meses (tendência);

β^0^, o intercepto antes da pandemia;

β^1^, a variação de Y_i_ em função do tempo;

β^2^, o impacto sobre a incidência de violência interpessoal com o início da pandemia; e

β^3^, a variação de Y_i_ somada a β^1^ em função do tempo durante a pandemia.

Estabeleceu-se o nível de significância de 5% para todos os testes estatísticos. Os dados foram analisados utilizando-se o software Stata 12.

## Resultados

Durante os nove anos estudados, foram registradas 268.337 notificações de violência interpessoal. Na análise, 70,7% de vítimas eram do sexo feminino, resultado que se repetiu para todos os ciclos de vida. Destacou-se a proporção de incompletude do campo de 9,2% em relação à raça/cor da pele. Entre as fichas com essa informação, 45,6% de vítimas de violência interpessoal foram as pessoas pardas (Tabela 1). 

**Tabela 1:** Frequências absolutas (n) e relativas (%) das notificações de violência interpessoal em cada ciclo de vida, por sexo, raça/cor da pele e tipo. Minas Gerais, 2014-2022 (n=268.337)

	Crianças	Adolescentes	Adultos	Pessoas idosas	Total
	n (%)	n (%)	n (%)	n (%)	n (%)
**Sexo **					
Masculino	9.444 (39,7)	18.617 (33,9)	41.098 (24,3)	9.398 (45,3)	78.557 (29,3)
Feminino	14.365 (60,3)	36.292 (66,1)	127.669 (75,7)	11.416 (54,9)	189.742 (70,7)
**Raça/cor da pele**					
Branca	7.140 (30,0)	16.202 (29,5)	54.931 (32,5)	8.517 (40,9)	86.790 (32,3)
Preta	2.296 (9,6)	6.343 (11,6)	20.462 (12,1)	2.390 (11,5)	31.491 (11,7)
Parda	11.117 (46,7)	25.994 (47,3)	76.930 (45,6)	8.175 (39,3)	122.216 (45,6)
Outras	325 (1,4)	587 (1,1)	2.027 (1,2)	213 (1,0)	3.152 (1,2)
Sem informações	2.937 (12,3)	5.791 (10,5)	14.438 (8,6)	1.522 (7,3)	24.688 (9,2)
Total	23.815 (100,0)	54.917 (100,0)	168.788 (100,0)	20.817 (100,0)	268.337 (100,0)
**Tipo de violência **					
**Violência física **					
Feminino	5.696 (27,9)	25.388 (48,6)	112.631 (61,7)	7.428 (43,2)	151.143 (55,5)
Masculino	5.243 (39,9)	15.922 (68,5)	38.136 (77,0)	7.318 (58,1)	66.619 (67,6)
**Violência psicológica/moral **					
Feminino	3.982 (19,5)	12.179 (23,3)	50.922 (27,9)	5.138 (29,9)	72.221 (26,5)
Masculino	2.518 (19,1)	3.844 (16,5)	7.682 (15,5)	2.483 (19,7)	16.527 (16,8)
**Violência sexual **					
Feminino	7.739 (37,9)	10.923 (20,9)	7.930 (4,4)	470 (2,7)	27.062 (9,9)
Masculino	2.587 (19,7)	1.232 (5,3)	437 (0,9)	43 (0,3)	4.299 (4,4)
**Violência por negligência/abandono **					
Feminino	2.178 (10,7)	1.154 (2,2)	842 (0,5)	2.406 (14,0)	6.580 (2,4)
Masculino	2.166 (16,5)	853 (3,7)	521 (1,1)	1.521 (12,1)	5.061 (5,1)
**Outras viol^ê^nciasa **					
Feminino	835 (4,1)	2.583 (5,0)	10.112 (5,5)	1.752 (10,2)	15.282 (5,6)
Masculino	643 (4,9)	1.384 (6,0)	2.749 (5,6)	1.236 (9,8)	6.012 (6,1)
**Total dos tipos de violências li^s^tadasb **					
Feminino	20.430 (100,0)	52.227 (100,0)	182.437 (100,0)	17.194 (100,0)	272.288 (100,0)
Masculino	13.157 (100,0)	23.235 (100,0)	49.525 (100,0)	12.601 (100,0)	98.518 (100,0)

^a^Soma dos seguintes tipos de violência: outra, tortura, financeira, legal, trabalho infantil, tráfico humano; ^b^Na mesma notificação, pode ser incluída mais de um tipo de violência sofrida.

A violência física foi a mais frequente entre as mulheres (55,5%), seguida por violência psicológica/moral (26,5%). Essa situação se repetiu para todos os ciclos de vida, com exceção das crianças do sexo feminino, em que a violência sexual ocupou o primeiro lugar (37,9%). A violência física também foi o tipo mais notificado (67,7%) entre os homens e seguiu o mesmo padrão em todos os momentos da vida. Destacaram-se as maiores frequências de negligência/abandono nos ciclos dos extremos da vida entre crianças e pessoas idosas (Tabela 1).

Em todos os ciclos de vida, houve mudança visual nas tendências de violência interpessoal após o início da pandemia de covid-19 (Figura 1). A comparação das médias e dos desvios-padrão das incidências nos períodos pré-pandêmicos e pandêmicos confirmou a queda em todos os ciclos de vida, tanto para o sexo masculino quanto feminino (p-valor>0,050 em todas as categorias) (Tabela 2).

**Tabela 2:** Média e desvio-padrão (DP) da incidência de notificações de violência interpessoal nos períodos pré-pandêmico e pandêmico (por 100 mil indivíduos), em cada ciclo de vida, por sexo. Minas Gerais, 2014-2022 (n=268.337)

	Período pré-pandêmico	Período pandêmico	
	Média (DP)	Média (DP)	Diferença (%)^a^
**Crianças **			
Feminino	10,98±1,59	9,76±1,58	11,1
Masculino	7,24±1,09	5,10±0,86	29,6
Total	9,07±1,19	7,38±1,11	18,6
Adolescentes			
Feminino	25,15±2,67	17,54±2,18	30,3
Masculino	12,87±1,74	6,87±1,09	35,6
Total	18,88±1,95	12,08±1,33	36,0
Adultos			
Feminino	22,45±1,88	15,86±1,70	29,4
Masculino	7,04±0,72	5,49±0,94	22,0
Total	14,74±1,21	10,66±1,27	27,7
**Pessoas idosas **			
Feminino	7,80±1,12	4,66±0,54	40,3
Masculino	7,68±0,99	4,57±0,52	40,5
Total	7,75±0,91	4,62±0,42	40,4

^a^Realizado teste t para comparação das médias, p-valor>0,050 em todas as categorias.

**Figura 1:** Tendência temporal da incidência mensal de violência interpessoal (por 100 mil habitantes), por ciclo de vida. Minas Gerais, 2014-2022 (n=268.337)

O modelo linear ajustado de Prais-Winsten permitiu verificar duas dimensões da variável incidência média de violência interpessoal: nível e tendência. A pandemia da covid-19 se comportou como uma segmentação temporal sobre a incidência de violência interpessoal, permitindo comparar o período pré-pandêmico e pandêmico nas dimensões supracitadas. No período de pré-pandemia de covid-19, observou-se tendência crescente na incidência de violência interpessoal contra crianças do sexo feminino (0,03 notificações por 100 mil habitantes; p-valor<0,050), enquanto para adolescentes do sexo masculino (-0,03 casos por 100 mil habitantes; p-valor<0,050) e pessoas idosas (-0,02 casos por 100 mil habitantes; p-valor<0,050) a tendência foi decrescente. Para os demais ciclos de vida e sexo, a incidência de violência interpessoal apresentou tendência estacionária (Tabela 3).

**Tabela 3:** Coeficientes de inclinação e intervalos de confiança de 95% (IC95%) das incidências de violência interpessoal nos períodos pré-pandêmico e pandêmico de covid-19 em cada ciclo de vida, por sexo. Minas Gerais, 2014-2022 (n=268.337)

Ciclo de vida	Período pré-pandêmico		Período pandêmico	
	​​𝜷​ 1​​​^a^ (IC95%)	Tendência	​​𝜷​ 2​​​^b^ (IC95%)	​​𝜷​ 3​​​^c^ (IC95%)
Crianças				
Feminino	0,03 (0,01; 0,05)^d^	Crescente	-2,10 (-3,78; -0,43)^d^	-0,03 (-0,12; 0,06)
Masculino	-0,014 (-0,03; 0,01)	Estacionária	-1,60 (-2,85; -0,36)^d^	-0,01 (-0,08; 0,06)
Total	0,0085 (-0,01; 0,03)	Estacionária	-1,85 (-3,26; -0,44)^d^	-0,02 (-0,10; 0,06)
Adolescentes				
Feminino	0,02 (-0,02; 0,05)	Estacionária	-8,55 (-11,30; -5,81)^d^	0,02 (-0,13; 0,17)
Masculino	-0,03 (-0,06; -0,01)^d^	Decrescente	-5,21 (-6,88; -3,55)^d^	0,06 (-0,03; 0,15)
Total	-0,01 (-0,04; 0,02)	Estacionária	-6,72 (-8,78; -4,67)^d^	0,03 (-0,08; 0,15)
Adultos				
Feminino	-0,00 (-0,04; 0,03)	Estacionária	-5,02 (-7,41; -2,63)^d^	-0,09 (-0,22; 0,05)
Masculino	-0,01 (-0,02; 0,00)	Estacionária	-1,02 (-1,94; -0,10)^d^	-0,01 (-0,06; 0,05)
Total	-0,01 (-0,03; 0,02)	Estacionária	-2,95 (-4,54; -1,36)^d^	-0,05 (-0,14; 0,04)
**Pessoas idosas **				
Feminino	-0,02 (-0,03; -0,00)^d^	Decrescente	-2,44 (-3,52; -1,36)^d^	0,01 (-0,05; 0,07)
Masculino	-0,02 (-0,03; -0,01)^d^	Decrescente	-2,26 (-3,08; -1,44)^d^	-0,01 (-0,05; 0,04)
Total	-0,02 (-0,03; -0,01)^d^	Decrescente	-2,35 (-3,18; -1,53)^d^	0,00 (-0,04; 0,05)

^a^Tendência da série no período pré-pandemia de covid-19; ^b^Medida do impacto imediato da pandemia de covid-19, o quanto a incidência média de violência interpessoal modificou (- para menos; + para mais); ^c^Medida de variação da tendência durante a pandemia. A tendência durante a pandemia deve ser interpretada como a soma de β_1_+β_3_; ^d^dp-valor<0,050.

No período pandêmico de covid-19, observou-se queda das notificações de violência interpessoal em todos os ciclos de vida, ou seja, uma mudança de nível na incidência de violência interpessoal, representada na tabela pelo coeficiente ​β​_2_ negativo (deslocamento do ​β​_0_; p-valor<0,050). As quedas mais acentuadas foram nas categorias adolescentes (-8,55 casos por 100 mil habitantes; p-valor<0,050) e adultos (-5,02 casos por 100 mil habitantes; p-valor<0,050), ambas no sexo feminino. A queda menos expressiva ocorreu no subgrupo homens adultos (-1,02 casos por 100 mil habitantes).

A queda nas notificações se manteve durante o período da pandemia de covid-19, uma vez que as tendências não sofreram alterações estatisticamente significativas em nenhum dos ciclos de vida (​β​_3_ não significativo), independentemente da tendência pré-pandemia (Tabela 3).

## Discussão

Este estudo teve como objetivo caracterizar as notificações de violência interpessoal e analisar sua tendência temporal entre 2014 e 2022, assim como o impacto da pandemia de covid-19 sobre esse agravo em Minas Gerais. Os achados indicaram que a maior proporção das notificações foi de violências que ocorreram contra mulheres e pessoas pardas. O tipo de violência mais relatada é a violência física, tanto para homens quanto para mulheres, em todos os ciclos de vida. Destacou-se a exceção para crianças do sexo feminino que sofrem violência sexual, mais que qualquer outro tipo. O início da pandemia de covid-19 marcou uma modificação da tendência temporal das incidências mensais de violência interpessoal, o que reduziu a notificação desse agravo em maior ou menor grau em todos os ciclos de vida. 

A violência contra as mulheres ganha forma em sociedades patriarcais, que as tolhem nos diferentes âmbitos da vida, dificultando seu empoderamento e sua autonomia. Essa situação perpetua e naturaliza a visão da mulher como ser inferior e mais frágil em relação ao homem, tornando mais vulnerável a episódios violentos [19]. A maior proporção de vítimas pardas demonstra o caráter interseccional da violência, em que gênero, raça/cor da pele, status socioeconômico e entre outras vulnerabilidades interagem fortalecendo o ciclo da violência [20]. No Brasil, em 2021, pessoas pretas e pardas, analisadas em conjuntos, apresentaram risco de morte violenta duas a três vezes maior que o de pessoas brancas [4,21].

O tipo de violência a que os indivíduos estão sob o risco se modifica de acordo com o sexo e o ciclo de vida. A violência física atravessa os ciclos de vida de forma diferente. Enquanto mulheres são vítimas principalmente de violência intradomiciliar, notadamente de parceria íntima, homens estão expostos à violência em ambientes externos, muitas vezes relacionados a comportamentos de risco como álcool e drogas, busca por status e poder [1]. A violência sexual tem um caráter de gênero marcante, sendo as mulheres as principais vítimas. A objetificação da mulher, aliada ao senso de pertencimento e domínio, características de uma sociedade machista como a do Brasil, faz com que elas sejam vulnerabilizadas desde a infância [19].

Neste estudo, as maiores frequências de negligência/abandono foram observadas nos ciclos dos extremos da vida, o que corrobora a hipótese de que o desequilíbrio de poder nas relações está entre as causas da violência interpessoal [22]. Crianças e pessoas idosas são, na maioria das vezes, dependentes dos cuidados de terceiros para a realização de atividades diárias, além da dependência financeira que pode coexistir [22,23]. Soma-se a isso o fato de que a busca por assistência médica e a denúncia de agressão nesse subgrupo é limitada. Ainda, os episódios violentos podem ser negados pelas próprias vítimas, por medo da repercussão intrafamiliar e social que ela possa causar [19]. Reforça-se a necessidade de políticas públicas voltadas para a prevenção de violências, com estratégias perenes que busquem, para além de punir, educar para uma cultura de paz e uma sociedade com igualdade de direitos. 

Diversos estudos sobre violência, que abarcaram o período da pandemia de covid-19, têm demonstrado que essa circunstância modificou a tendência de casos de violência. Os resultados variam de acordo com a metodologia [24], a população de estudo [22,25] e a fonte dos dados [26]. Neste estudo, foi possível observar que, em todos os ciclos de vida, tanto em homens quanto em mulheres, as médias de incidência de violência interpessoal pré-pandemia de covid-19 eram superiores às médias durante a pandemia (Tabela 2). Ao menos duas vias podem explicar esses resultados. De um lado, pode ter havido real queda dos casos de violência, embora no Brasil a violência contra mulheres tenha crescido de 9,8 para 19,2 por 100 mil mulheres de 2020 para 2023 [20]. Por outro, pode ter havido mudanças no perfil de notificação dos casos durante a pandemia.

Ao longo do período crítico da pandemia, a orientação era de que os serviços de saúde fossem acessados apenas em casos urgentes. Houve reestruturação das unidades de saúde, sobretudo as de atenção básica, para o atendimento às demandas da nova doença [27]. No caso das violências que ocorreram no âmbito domiciliar, as medidas de distanciamento social facilitaram o controle do agressor sobre as vítimas, o que dificultou acessar a sua rede de apoio e buscar por ajuda [22]. Em relação a crianças e adolescentes, o fechamento das instituições de ensino, como escolas e creches, também pode ter atuado como dificultador da detecção de vítimas de violência infantil pelos profissionais da educação, uma vez que esses trabalhadores desempenham papel importante na identificação de casos suspeitos [22,27]. 

Já para as violências que ocorrem fora do domicílio, que acometem principalmente jovens e homens adultos, o distanciamento social imposto durante a pandemia de covid-19 pode ter contribuído para reduzir a violência física [24]. A interação entre esses fatores, aliada ao fato de não haver mudanças nas políticas de proteção às vítimas, ou mudanças culturais expressivas, reforça a possibilidade de que a subnotificação dos casos tenha sido determinante para a queda nas incidências de violência interpessoal. 

Neste estudo, demonstraram-se tendências inalteradas de incidência da violência interpessoal durante o período da pandemia. Ou seja, mesmo após o afrouxamento do distanciamento social, com a adequação dos serviços de saúde e das instituições de ensino às novas demandas, reabertura do comércio etc., os subgrupos que apresentavam tendência de incidência anterior crescente ou decrescente mantiveram tais tendências inalteradas, só que em níveis mais baixos. Esse resultado demonstra que os impactos podem ser duradouros e a pandemia ser mais um agente invisibilizador da violência.

Este estudo utilizou dados secundários obtidos, majoritariamente, nos serviços de saúde. Dessa forma, não foi possível garantir que a coleta foi padronizada, nem que se tratou da totalidade dos casos de ocorrência de violência interpessoal. Admite-se que duplicidades possam ter ocorrido. Adicionalmente, a variável raça/cor da pele apresentou incompletude de 7% a 12%, dependendo do ciclo de vida, portanto os dados relativos a ela devem ser interpretados com cautela. Apesar das limitações, inerentes à fonte dos dados, o Sistema de Informação de Agravos de Notificação tem sido consistentemente utilizado em estudos epidemiológicos e demonstrado boa validade [26,28,29]. 

O delineamento do estudo não permitiu inferir causalidade, mas elucidou associações que subsidiam o levantamento de hipóteses causais a serem confirmadas em estudos posteriores. A generalização dos resultados deve ser realizada com cautela, uma vez que foram incluídas apenas notificações ocorridas em Minas Gerais, que podem não representar a população brasileira. A inclusão de variáveis de controle (existência de políticas públicas, diferenças locais de acesso à saúde e desigualdades regionais) poderia refinar o modelo, sendo, portanto, uma possibilidade de estudos futuros. Além disso, estudos que abordem qualitativamente como a pandemia impactou as dinâmicas familiares e comunitárias podem contribuir para o entendimento das vias de manutenção da violência no curso de vida, bem como subsidiar discussões de políticas públicas de enfrentamento a esse agravo.

Conclui-se que a pandemia de covid-19 impactou as notificações de violência de maneira heterogênea nos diferentes ciclos de vida, o que evidenciou padrões divergentes de acordo com o sexo e a faixa etária. Isso demonstrou que a violência interpessoal atravessa os diferentes ciclos de vida de maneira diversa, tornando os subgrupos populacionais mais vulneráveis a determinados tipos de violência.

A pandemia de covid-19 foi capaz de alterar os padrões da incidência de violência interpessoal, calculadas a partir das notificações de violência interpessoal neste estudo. Tais achados corroboraram a importância de os serviços de saúde estarem preparados para a ocorrência de emergências em saúde pública. Além disso, elucidaram a necessidade de políticas públicas que fortaleçam as vias de denúncia, os mecanismos de proteção às vítimas, considerando as especificidades de cada ciclo de vida.

## Data Availability

O banco de dados e os códigos de análise utilizados na pesquisa estão disponíveis em: https://doi.org/10.48331/SCIELODATA.UMERVN [30].
